# An insight into thermal properties of BC_3_-graphene hetero-nanosheets: a molecular dynamics study

**DOI:** 10.1038/s41598-021-02576-6

**Published:** 2021-11-29

**Authors:** Maryam Zarghami Dehaghani, Fatemeh Molaei, Farrokh Yousefi, S. Mohammad Sajadi, Amin Esmaeili, Ahmad Mohaddespour, Omid Farzadian, Sajjad Habibzadeh, Amin Hamed Mashhadzadeh, Christos Spitas, Mohammad Reza Saeb

**Affiliations:** 1 Center of Excellence in Electrochemistry, School of Chemistry, College of Science, University of Tehran, Tehran, Iran; 2 Mining and Geological Engineering Department, The University of Arizona, Arizona, USA; 3 Department of Physics, University of Zanjan, 45195-313 Zanjan, Iran; 4 Department of Nutrition, Cihan University-Erbil, Kurdistan Region, Erbil, Iraq; 5 Department of Phytochemistry, SRC, Soran University, KRG, Erbil, Iraq; 6 Department of Chemical Engineering, College of the North Atlantic—Qatar, 24449 Arab League St, PO Box 24449, Doha, Qatar; 7 College of Engineering and Technology, American University of the Middle East, Egaila, Kuwait; 8 Mechanical and Aerospace Engineering, School of Engineering and Digital Sciences, Nazarbayev University, Nur-Sultan, 010000 Kazakhstan; 9 Department of Chemical Engineering, Amirkabir University of Technology (Tehran Polytechnic), Tehran, Iran; 10 Department of Polymer Technology, Faculty of Chemistry, Gdańsk University of Technology, G. Narutowicza 11/12, 80-233 Gdańsk, Poland

**Keywords:** Graphene, Theory and computation

## Abstract

Simulation of thermal properties of graphene hetero-nanosheets is a key step in understanding their performance in nano-electronics where thermal loads and shocks are highly likely. Herein we combine graphene and boron-carbide nanosheets (BC3N) heterogeneous structures to obtain BC3N-graphene hetero-nanosheet (BC3GrHs) as a model semiconductor with tunable properties. Poor thermal properties of such heterostructures would curb their long-term practice. BC_3_GrHs may be imperfect with grain boundaries comprising non-hexagonal rings, heptagons, and pentagons as topological defects. Therefore, a realistic picture of the thermal properties of BC_3_GrHs necessitates consideration of grain boundaries of heptagon-pentagon defect pairs. Herein thermal properties of BC_3_GrHs with various defects were evaluated applying molecular dynamic (MD) simulation. First, temperature profiles along BC_3_GrHs interface with symmetric and asymmetric pentagon-heptagon pairs at 300 K, ΔT = 40 K, and zero strain were compared. Next, the effect of temperature, strain, and temperature gradient (ΔT) on Kaptiza resistance (interfacial thermal resistance at the grain boundary) was visualized. It was found that Kapitza resistance increases upon an increase of defect density in the grain boundary. Besides, among symmetric grain boundaries, 5–7–6–6 and 5–7–5–7 defect pairs showed the lowest (2 × 10^–10^ m^2^ K W^−1^) and highest (4.9 × 10^–10^ m^2^ K W^−1^) values of Kapitza resistance, respectively. Regarding parameters affecting Kapitza resistance, increased temperature and strain caused the rise and drop in Kaptiza thermal resistance, respectively. However, lengthier nanosheets had lower Kapitza thermal resistance. Moreover, changes in temperature gradient had a negligible effect on the Kapitza resistance.

## Introduction

Graphene, sp^2^-hybridized carbon, with honeycomb crystal lattice, has received considerable attention from its first production due to possessing outstanding features such as superior Young’s modulus (1000–1500 GPa)^1^, high adsorption capacity^2^, excellent thermal conductivity (5000 W m^−1^ K^−1^)^3–5^, desirable electrical conductivity (2000s m^−1^)^6^, and large surface area (2630 m^2^ g^−1^)^7^. Therefore, graphene supports various fields such as biological, environmental, and engineering applications^8–13^. On the other hand, graphene suffers from zero bandgap, limiting its usefulness as a semiconductor or transistor. Therefore, the inevitable hybridization of graphene with non-zero bandgap nanosheets such as boron-nitride (BN)^14–19^, boron-carbide (BC_3_)^20,21^, nitrogen-carbide (C_3_N)^22^, silicone-germanium (SiGe)^23^, silicone-carbide^24^, and beryllium-oxide (Be-O)^25,26^ form planar heterostructures to produce nanosheets with tunable electrical, thermal, and mechanical properties^27^.

The boron-carbide nanosheets (BC_3_NSs), as a carbonaceous semiconductor, possess a bandgap ranging from 0.4 to 0.7 eV. Concerning the theoretical research on physical properties of the BC_3_NS, such nanosheets have room temperature Young’s modulus, tensile strength, and thermal conductivity of 256, 29.0 N m^−1^, 410 W m^−1^ K^−1^, respectively^28^. Moreover, the integration of BC_3_NSs with a similar lattice structure with graphene forms in-plane heterostructures composed of BC_3_NS and graphene with tunable properties. However, BC_3_NS-graphene hetero-nanosheets (BC_3_GrHs) may not be ideally acquired as a perfect lattice because of topological defects at the juncture of grain boundaries containing non-hexagonal rings, i.e., heptagons and pentagons. Typically, more difficulties may also be brought about at elevated temperatures, such that the inter-particle distance would be increased because of attenuated molecular motions. Thus, the boundary regions are unavoidably under more stress and may damage. In fact, defects weaken the physical and mechanical properties of BC_3_NS under overheating conditions^22^.

Due to the potential overheating of the heterostructure nanosheets applied in nano-electronics and storage devices, understanding the thermal transport along such nanodevices is significant to estimate their lifetime and performance. In this regard, several theoretical studies attempted to predict the thermal properties of the heterostructure nanosheets. For example, Li *et al.*^29^ studied the thermal properties of heterostructure nanosheet composed of graphene and hexagonal BN using molecular dynamics (MD) simulation. They modeled several graphene-BN heterostructures having grain boundaries with various defect densities of heptagons and pentagons. It was reported that the interfacial conductance decreased from 5.4 × 10^10^ to 3.0 × 10^10^ W m^−2^ K^−1^ upon an increase of mismatch rotation angle of grains from 10° to 25°, and subsequently increasing the density of defects in grain boundaries. Mortazavi *et al.*^30^ verified thermal conductivity in graphene-borophene heterostructure nanosheets using MD simulation and density functional theory (DFT). They reported that the thermal conductance in grain boundaries was not intensively affected by the topology of defects so that the thermal conductance varied in the range of 0.26–36 GW m^−2^ K^−1^. The effects of vacancy defects in grain boundary and strain on interfacial thermal conductivity of graphene-BN heterostructure nanosheet were shown in the theoretical works done by Son *et al*.^31^. However, vacancy defects were found as the leading cause of enhanced interfacial thermal conductance; however, with lower inherent thermal conductivity.

Moreover, the increase of the strain caused a decrease in interfacial thermal conductance. Yao *et al.*^32^ reported the effects of vacancy defect, strain, and temperature fields on the interfacial thermal transport of grain boundary in graphene-BN heterostructure by MD simulation, notifying enhanced heat transfer from grain boundaries at higher temperatures. At the same time, an inverse trend was observed once the size of the defect increased. Moreover, the compression strain could not significantly affect the interfacial thermal conductivity. Sadeghzadeh *et al*.^33^, studied the mechanical properties of defective hybrid C_3_N-BC_3_ nanosheets.

So far, parameters affecting the thermal conductivity and temperature profile of the heterostructure nanosheets, such as defect density in the grain boundary, strain, and temperature, are studied. However, there is no image of thermal properties of BC_3_GrHs heterostructure, neither theoretically nor experimentally. Theoretical investigation of such phenomenon would help deepen understanding of the performance of such complex conductive materials needed for future developments in electronics, military, and aerospace applications. In the present work, we demonstrated the temperature profile along BC_3_GrHs together with visualization of the interfacial thermal resistance (Kapitza resistance) in the grain boundaries using MD simulations. The temperature profiles of BC_3_GrHs with grain boundaries (containing symmetric and asymmetric pentagons and heptagons with various defect concentrations) were first tested at 300 K and ΔT = 40 K. Next, the effects of temperature changes, temperature gradient (ΔT), and strain on Kapitza resistance of BC_3_GrHs having grain boundaries were unveiled and discussed.

## Simulation method

**Figure 1:**
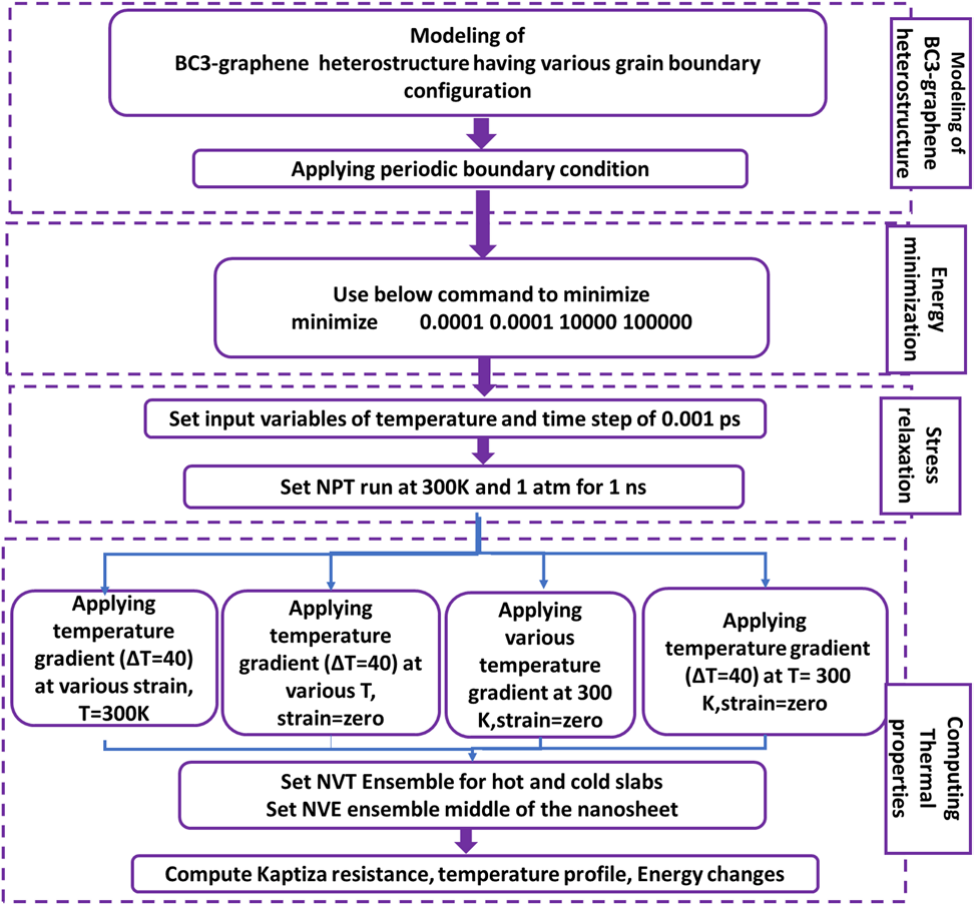
Flowchart of the overall simulation stages, including modeling, energy minimization, stress relaxation and calculation of thermal properties.

In this research, MD simulation by open-source software Large-Scale Atomic/Molecular Massively parallel Simulator (LAMMPS)^34^ was applied to investigate the thermal properties of BC_3_GrHs having grain boundaries containing symmetric and asymmetric pentagon-heptagon defect pairs with various defect concentrations. The bonding interactions of carbon–carbon and carbon-Boron were modeled using the optimized Tersoff potential^35^. Figure 1 shows the flowchart of simulation stages, including the structure and framework of the modeling, along with energy minimization, stress relaxation, and computation of thermal properties.

**Figure 2:**
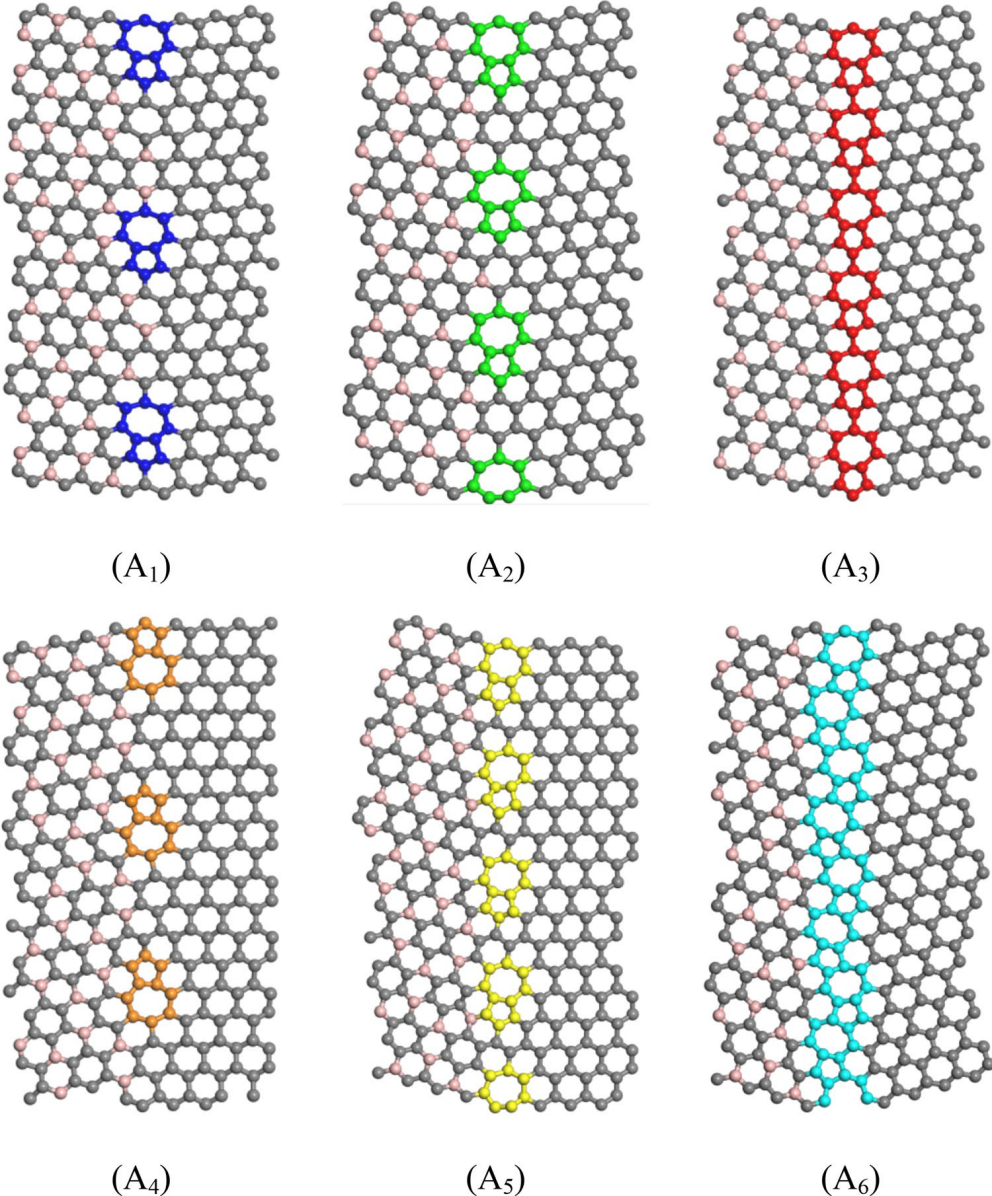
Top views of the atomic configuration of six different grain boundaries consisting of pentagon-heptagon defect pairs with different defect concentrations: (A_1_) 5–7–6–6–s, (A_2_) 5–7–6–s, (A_3_) 5–7–5–7–s, (A_4_) 5–7–6–6–a, (A_5_) 5–7–6–a, and (A_6_) 5–7–5–7–a. “s” and “a” denote as symmetric and asymmetric, respectively.

Figure 2 shows the atomic configuration of the symmetric and asymmetric grain boundaries containing pentagon-heptagon pairs. Notably, perfect grain boundaries in BC_3_GrHs consist of hexagons. As in Fig. 2, A1, A2, A3 schematics are related to the symmetric grain boundaries having ring series of pentagon-heptagon-hexagon-hexagon (5–7–6–6), pentagon-heptagon-hexagon (5–7–6), and pentagon-heptagon- pentagon-heptagon (5–7–5–7), respectively. A_4_, A_5_, and A_6_ schematics correspond to the asymmetric grain boundaries having ring series of pentagon-heptagon-hexagon-hexagon (5–7–6–6), pentagon-heptagon-hexagon (5–7–6), and pentagon-heptagon- pentagon-heptagon (5–7–5–7). In other words, 5–7–6–6 configurations (A_1_, A_4_), 5–7–5–7 configurations (A_3_, A_6_) have the lowest and highest defect density, respectively, along grain boundaries. Moreover, two, one, and zero hexagonal rings separated two 5–7 defect pairs in A_1_ (or A_4_), A_2_ (or A_5_), and A_3_ (or A_6_) configurations, respectively.

The width and length of all BC_3_GrHs were 10 and 30 nm, respectively. The periodic boundary conditions were considered in both X and Y directions.

**Figure 3:**
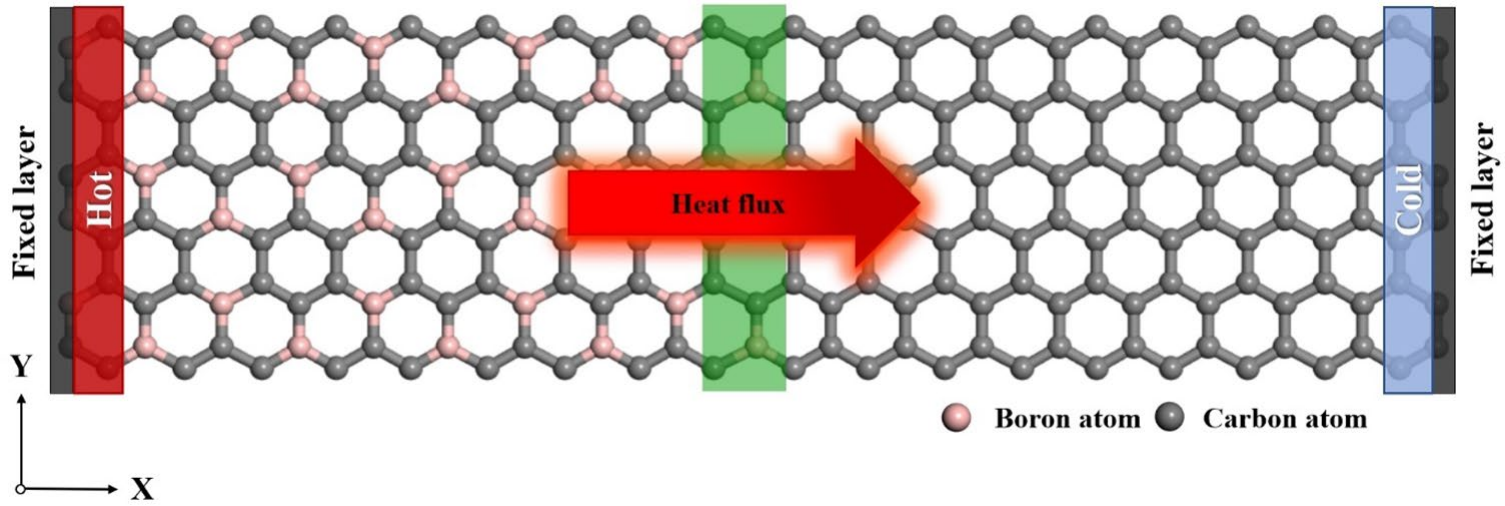
MD setup for evaluating thermal properties of BC_3_GrHs. Gray and pink balls denote carbon and boron atoms, respectively. The periodic boundary condition is applied along the X and Y directions. The heat flux is in the X-direction. The green, red, and blue regions show the grain boundary, hot slab, and cold slab, respectively. The visualization was obtained by means of visual MD (VMD) simulation (Ver. 1.9.3, https://www.ks.uiuc.edu/Development/Download/download.cgi?PackageName=VMD).

Figure 3 represents MD setup for computing the temperature gradient along the heat transfer direction and Kapitza resistance in grain boundaries. For these purposes, the nanosheet models were divided into 30 slabs along the X-direction. Atoms present in the left and right edges of nanosheets were fixed. Applying the NVT ensemble (Nose–Hoover thermostat method), the temperature at the hot and cold slabs were defined as $T + \Delta T/2$ and $T - \Delta T/2$, respectively. The heat flux (*J*_*x*_) along the X direction in nanosheets is expressed as below^36^:

1 $$J_{x} = \frac{{{\raise0.7ex\hbox{${dE}$} \!\mathord{\left/ {\vphantom {{dE} {dt}}}\right.\kern-\nulldelimiterspace} \!\lower0.7ex\hbox{${dt}$}}}}{A}$$

where $A_{c}$ is the cross-section area of the nanosheet, *t* is simulation time, and *E* is accumulated energy. The thickness of nanosheets was considered 3.62 Å, which was the average value of van der Waals diameters of carbon (3.4 Å) and boron (3.84 Å)^37^.

Furthermore, it is possible to establish a relationship between the heat flux (*q*_*x*_), and the temperature drop in grain boundary $\left( {\Delta T_{GB} } \right)$ as following equation in which $R_{k}$ is the interfacial thermal resistance in grain boundary (Kapitza resistance)^38^:

2 $$R_{k} = \frac{{\Delta T_{GB} }}{{J_{x} }}$$

After the calculating Kapitza resistance of grain mentioned above boundaries in BC_3_GrHs, and obtaining temperature profile along BC_3_GrHs having various grain boundaries at T = 300 K and ΔT = 40 K, the effect of temperature increase (from 300 to 650 K), changes of temperature gradient (ΔT in range of 20–55 K), and applying strain (from 0.01 to 0.08%) on Kapitza resistance were verified.

## Results and discussion

Due to the inevitable presence of the grain boundaries consisting of pentagon-heptagon pairs in the structure of BC_3_GrHs, which cause the creation of Kapitza resistance and affect the temperature profile and energy changes, in the following sections, we placed the focus on verifying the mentioned thermal properties.

### Temperature profile and heat current evaluation in BC_3_GrHs

**Figure 4:**
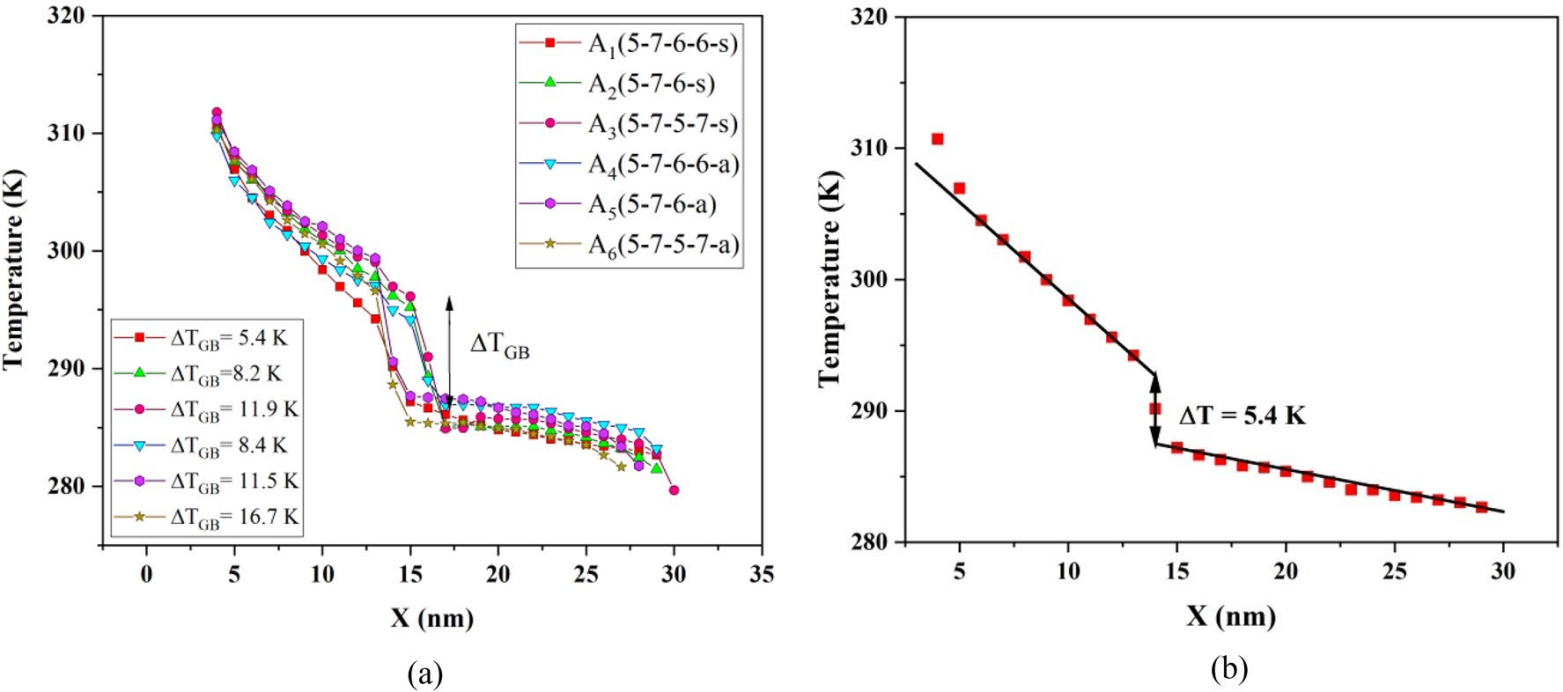
(**a**)The steady-state one-dimension temperature profiles for BC_3_GrHs having grain boundaries-type A_1_, A_2_, A_3,_ A_4_, A_5_, A_6_ along X direction with the same length of 30 nm at T = 300 K and ΔT = 40 K. ΔT_GB_ is the temperature drop in the grain boundary, (**b**) the linear fitting performed on the temperature profile for BC_3_GrHs having grain boundary-type A_1_ to obtain temperature drop (ΔT) at the grain boundary.

To obtain more insight into the effect of grain boundary on the thermal properties of the BC_3_GrHs, the changes of temperature along nanosheets were determined. Figure 4a and b show the temperature profiles of the BC_3_GrHs having various types of grain boundaries (A_1_, A_2_, A_3_, A_4_, A_5_, and A_6_) along X direction at room temperature and temperature gradient of 40 K. The temperature profiles of all nanosheets having grain boundaries containing heptagon-pentagon pairs revealed discontinuity in the middle so that the temperature dropped dramatically in the grain boundary placed at X = 15 nm. It can be speculated that pentagons and heptagons in the grain boundaries act as topological defects, which assist phonon scattering in the middle of BC_3_GrHs, ending in a temperature drop (ΔT_GB_). Li *et al*.^29^ reported a similar nonlinearity in temperature profile for graphene-boron nitride heterostructure having 5–7 defects along the grain boundaries. In another MD simulation, Mayelifartash *et al.*^39^ reported a temperature drop at the C3N and BC3 nanosheets interface in the hybrid C_3_N-BC_3_ nanosheets.

Figure 4 also suggests an increase in temperature drop upon increasing the defect density in grain boundaries, which means that the BC_3_GrHs with 5–7 and 5–7–5–7 defect pairs show the lowest and highest temperature drop, respectively. This can be ascribed to enhanced phonon scattering in grain boundaries having higher defect concentration. Moreover, the temperature drop in non-symmetric grain boundaries was higher than that of symmetric grain boundaries at the specified defect concentration. A similar trend for temperature drop in the vicinity of grain boundaries, having mentioned pentagon-heptagon defect pairs, was reported for polycrystalline silicene by Khalkhali *et al*.^40^.

**Figure 5:**
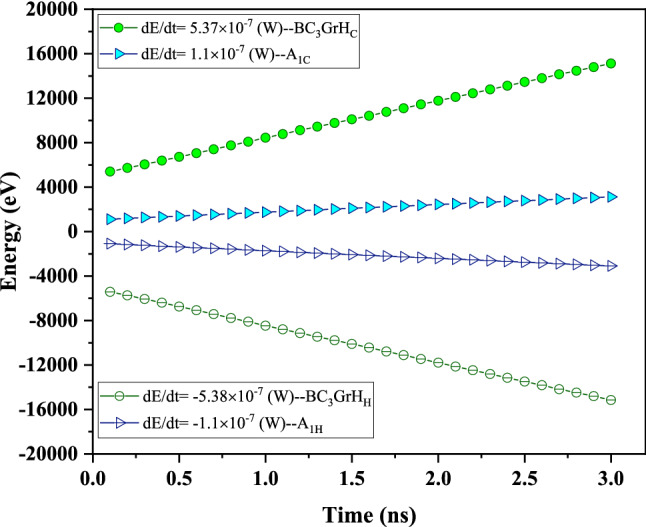
Change in the amounts of energy in the cold (C-coded symbols) and hot (H-coded symbols) slabs as a function of simulation time for BC_3_GrH having perfect grain boundary (hexagonal rings) and BC_3_GrH having grain boundary-type A_1_ (5–7–6–6–s), with the same lengths and width of 30 nm and 10 nm, respectively, at T = 300 K and ΔT = 40 K. Filled symbols indicate effluent of H slab, while hollow symbols indicate heat entered into the C slab.

Figure 5 compares the simulation outputs in terms of time-dependent energy of the hot and cold slabs in BC_3_GrH structure with grain boundaries of A_1_ (5–7–6–6–s) type, besides, BC_3_GrH with perfect grain boundary of hexagonal rings type at fixed T = 300 K and ΔT = 40 K. The figure also makes it possible to compare heat flux values $\left( {\frac{dE}{{dt}}} \right)$ of hot and cold slabs. Expectedly, a descending and an ascending linear trend in energy variation over simulation time are the cases for the hot and cold slabs of nanosheets, respectively. Furthermore, we can see that the absolute amounts of energy in the cold and hot zones are approximately equal, which means conservation of energy or model verification. The same trend of energy variation in the hot and cold slabs was observed in the polycrystalline BC3 nanosheets, which confirms that the total energy of each simulated system remained constant^41^. In another theoretical research performed by Yousefi *et al.*^42^, the calculation of the accumulated energy in the hot and cold slabs of nanoporous graphene revealed a similar trend.

The comparison between the rates of heat transfer or heat flux values, featured by the considerable difference between the slopes of energy variation plots (Fig. 5), makes evident that BC_3_GrH with a perfect grain boundary characteristic is more sensitive to the time compared to the corresponding structure with grain boundaries of type A_1_. A considerably higher rate of heat transfer in defect-free BC_3_GrH nanosheet is indicative of a significantly lower resistance against phonon transfer because of architecturally ordered hexagonal grain boundaries allowing diffusion of phonons. On the other hand, the non-uniform grain boundaries in A_1_ (5–7–6–6–s) polygonal BC_3_GrH nanosheet cause an additional resistance resulting from the collision between highly scattered phonons. Since the interface region at BC_3_-GrH juncture dissipates energy, and the phonon spectrum of atoms in both sides of the grain boundaries dissipates the energy, such a drop in the heat flux of non-uniform structures would be speculated^43^.

### Kapitza resistance of grain boundaries containing pentagons and heptagons in BC_3_GrHs

**Figure 6:**
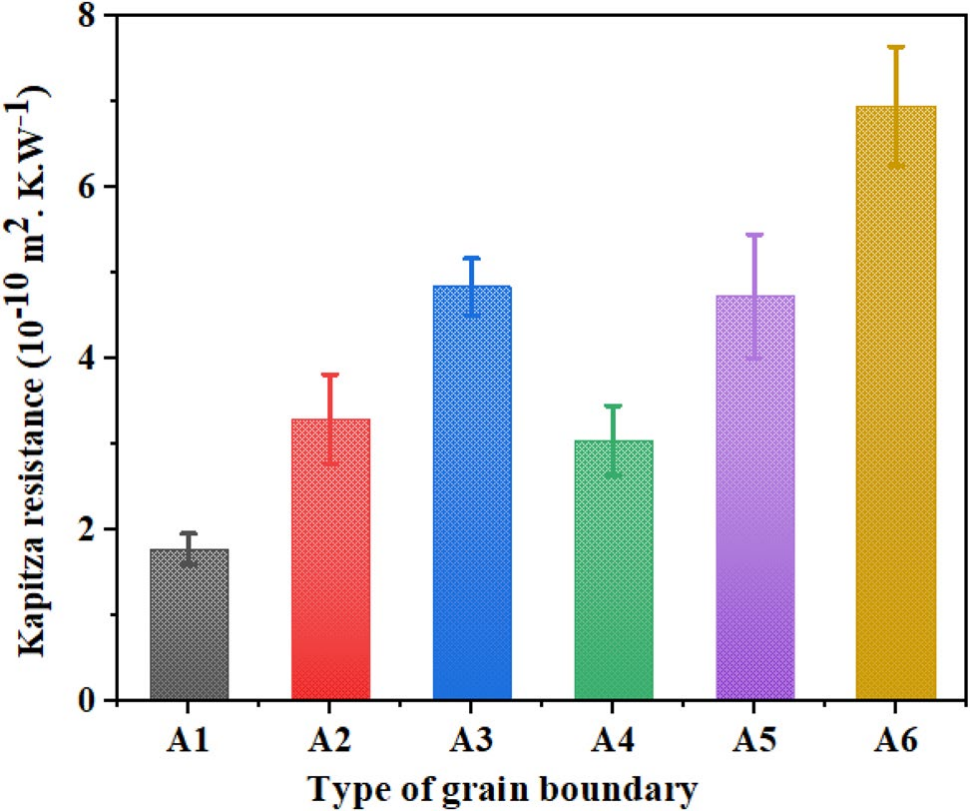
The interfacial thermal resistance (Kapitza resistance) of various constructed grain boundaries A_1_ (5–7–6–6–s), A_2_ (5–7–6–s), A_3_ (5–7–5–7–s), A_4_ (5–7–6–6–a), A_5_ (5–7–6–a), and A_6_ (5–7–5–7–a) in BC_3_GrHs at T = 300 K and ΔT = 40 K. “s” and “a” denote as symmetric and asymmetric.

After calculating the values of the heat current and temperature drop at grain boundaries, the interfacial thermal resistance (Kapitza resistance) values at six mentioned grain boundaries were compared. Figure 6 demonstrates the values of Kapitza resistance of grain boundaries A_1_ (5–7–6–6–s), A_2_(5–7–6–s), (A_3_) 5–7–5–7–s, A_4_ (5–7–6–6–a), A_5_ (5–7–6–a), and A_6_ (5–7–5–7–a) in BC_3_GrHs at T = 300 K and ΔT = 40 K. As can be observed, Kapitza resistance increased by increasing the defect concentration in grain boundaries so that A_1_ and A_3_ showed the lowest (2 × 10^–10^ m^2^ K W^−1^) and highest (4.9 × 10^–10^ m^2^ K W^−1^) values, respectively, among symmetric grain boundaries. For non-symmetric grain boundaries, the values of Kapitza resistance varied in the range of 3.1–7.2 × 10^–10^ m^2^ K W^−1^. As mentioned before, the grain boundary having a higher defect density caused more diverse lattice structures along the heat current direction between two grains. This caused the higher phonon scattering rate in the grain boundary, which acted as a barrier front heat flow and increased Kapitza thermal resistance.

Moreover, the non-symmetric grain boundaries showed higher Kapitza resistance than the symmetric one at specified defect concentration due to the higher phonon–phonon scattering. The observed increasing trend for Kapitza resistance by increasing defect density is in good agreement with the obtained results for MoS_2_ single-layer heterostructures reported by Mortazavi *et al*.^44^. In another work performed by Yao *et al*.^32^, the interfacial thermal conductivity of grain boundary in graphene-BN planar heterostructure decreased by increasing the percentage of vacancy defects at the interface. Likewise, Lio *et al*.^45^ showed the effect of defect density of grain boundary presented in bicrystal ZnO on the Kapitza thermal resistance, which revealed the same trend.

#### Effect of temperature on Kapitza resistance

**Figure 7:**
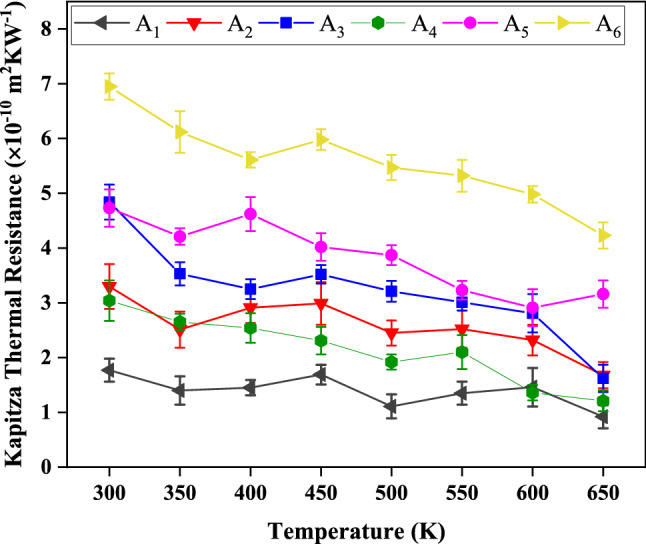
The changes in Kapitza thermal resistance of six constructed grain boundaries A_1_ (5–7–6–6–s), A_2_(5–7–6–s), A_3_ (5–7–5–7–s), A_4_ (5–7–6–6–a), A_5_ (5–7–6–a), and A_6_ (5–7–5–7–a) in BC_3_GrHs as a function of temperature at ΔT = 40 K and zero strain.

To evaluate the effect of elevated temperature on Kapitza resistance of grain boundaries, the values of Kapitza resistance of grain boundaries-type A_1_, A_2_, A_3_, A_4_, A_5_, and A_6_ at various temperatures of 350 K, 400 K, 450 K, 500 K, 550 K, 600 K, 650 K were computed. Figure 7 shows the alteration in Kapitza resistance of grain boundaries as a function of temperature at Δ*T* = 40 K and zero strain. As seen, the Kapitza resistance of all types of grain boundaries decreased by elevating temperature. For example, for A_6_ and A_3_, the Kapitza resistance decreased from the values of 7.2 × 10^–10^ and 4.9 × 10^–10^ m^2^ K W^−1^ to the values of 4.3 × 10^–10^ and 1.5 × 10^–10^ m^2^ K W^−1^ with decrement of about 40% and 69.3%, respectively by increasing the temperature from 300 to 650 K. It can be explained that the temperature enhancement caused the excitement of high-frequency phonons and subsequently simplification of phonon transmission. Therefore, the presence of phonons possessing higher energy facilitated the heat flux along the grain boundary and resulted in higher interfacial thermal conductivity and lower Kapitza resistance^46^. A similar effect of the temperature elevation on Kaptiza resistance was observed for graphene-boron nitride hetero-nanosheets in the works done by Eshkalak *et al*.^47^ and Liu *et al.*^48^.

#### Effect of temperature gradient (ΔT) on Kapitza resistance

**Figure 8:**
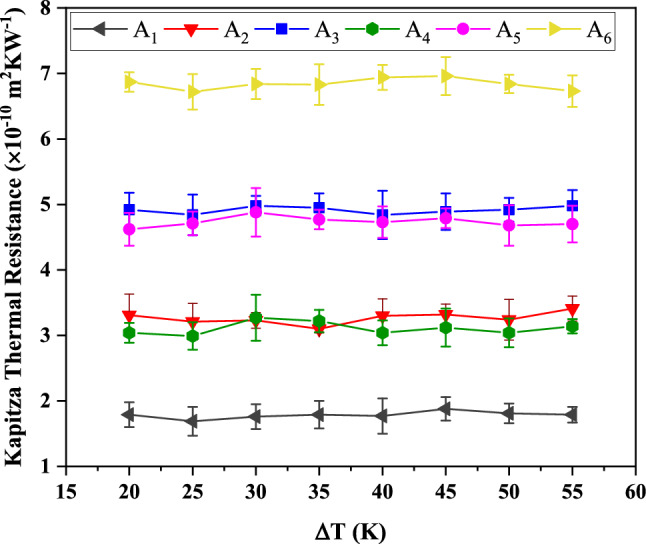
The changes in Kapitza thermal resistance of six constructed grain boundaries A_1_ (5–7-6–6-s), A_2_ (5–7-6-s), A_3_ (5–7–5–7–s), A_4_ (5–7–6–6–a), A_5_ (5–7–6–a), and A_6_ (5–7–5–7–a) in BC_3_GrHs as a function of ΔT at 300 K and zero strain.

The temperature gradient is considered as a determining parameter on heat current in BC_3_GrHs. However, to clarify its effect on Kapitza resistance of the grain boundaries, in this section, the variation in Kapitza resistance at various temperature gradients (ΔT) is verified. The changes in Kapitza thermal resistance of grain boundaries-type A_1_, A_2_, A_3_, A_4_, A_5,_ and A_6_ in BC_3_GrHs as a function of ΔT at 300 K and zero strain are depicted in Fig. 8. As seen, the values of Kapitza resistance of all grain boundaries vary in a narrow range, which confirms that the Kapitza resistance is independent of ΔT variation. According to the definition of the Kapitza resistance $$\left( {R_{k} = \frac{{\Delta T_{GB} }}{{\frac{{{\raise0.7ex\hbox{${dE}$} \!\mathord{\left/ {\vphantom {{dE} {dt}}}\right.\kern-\nulldelimiterspace} \!\lower0.7ex\hbox{${dt}$}}}}{A}}}} \right)$$, the change in ΔT caused the variation in both $$\Delta T_{GB}$$ and $${\raise0.7ex\hbox{${dE}$} \!\mathord{\left/ {\vphantom {{dE} {dt}}}\right.\kern-\nulldelimiterspace} \!\lower0.7ex\hbox{${dt}$}}$$ equally, which at least resulted in minor changes in the value of Kapitza resistance.

#### Effect of strain on Kapitza resistance

**Figure 9:**
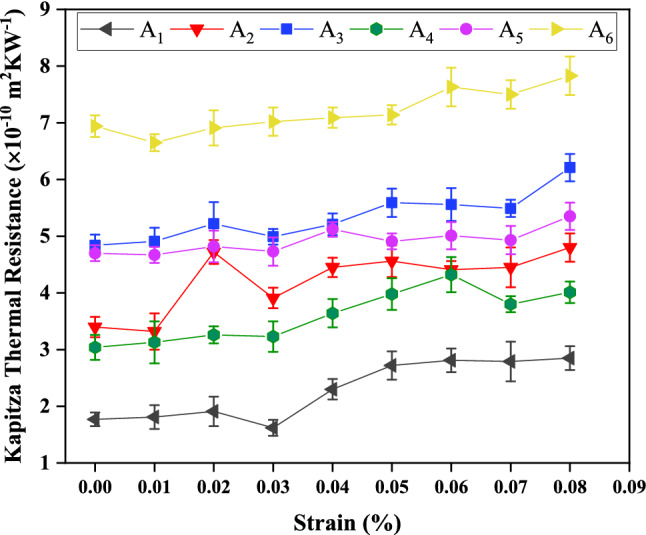
The changes in Kapitza thermal resistance of six constructed grain boundaries A_1_ (5–7-6–6-s), A_2_ (5–7-6-s), A_3_ (5–7–5–7–s), A_4_ (5–7–6–6–a), A_5_ (5–7–6–a), and A_6_ (5–7–5–7–a) in BC_3_GrHs as a function of strain at ΔT = 40 K and T = 300 K.

Due to the possible creation of mechanical strain in BC_3_GrHs, whether synthetic or intrinsically, it is important to understand the strain-thermal properties relationship. Figure 9 shows the alteration in Kapitza resistance of grain boundaries-type A_1_ to A_6_ as a function of strain at room temperature and ΔT = 40 K. It can be observed that the rise of the strain caused the increase in Kapitza resistance of all grain boundaries. For instance, the Kapitza resistance of grain boundaries type-A_3_ and A_6_ increased from the values of 4.9 × 10^–10^ and 6.94 × 10^–10^ m^2^ K W^−1^ to the values of 6.3 × 10^–10^ and 7.9 × 10^–10^ m^2^ K W^−1^ with an increment of about 22.22% and 13.8%, respectively. The increase of strain in the direction of heat current caused the increment of bond length and subsequently weakened atomic interaction.

Moreover, the phonon speed of atoms decreased by increasing the strain. These two outcomes arising from strain increment led to the decrease in Kapitza resistance. Eshkalak *et al*.^46^ reported a similar effect of strain on Kapitza resistance of grain boundary in C_3_N-graphene heterostructure.

#### Effect of Length on Kapitza resistance

**Figure 10:**
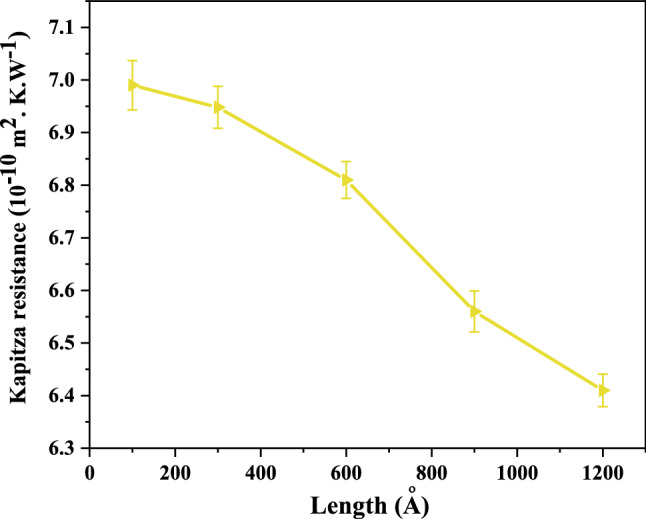
The changes in Kapitza thermal resistance of constructed grain boundaries A_6_ (5–7–5–7–a) in BC_3_GrHs function of nanosheet length at ΔT = 40 K and T = 300 K.

Another parameter that may affect the Kaptiza resistance is the length of the nanosheet as a scale of the length of the heat transfer path. Figure 10 shows the alteration of Kapitza thermal resistance of the constructed grain boundaries A_6_ (5–7–5–7–a) in the BC_3_GrHs as a function of the length of the nanosheet. The Kapitza thermal resistance in grain boundary decreased from the value of 6.99 × 10^–10^ m^2^ K W^−1^ to the value of 6.4 ⨯10^–10^ m^2^ K W^−1^ by increasing the length of the nanosheet from 100 to 1200 Å. A similar decreasing trend in Kapitza resistance of grain was observed in Azizi *et al*.^49^ by increasing the nanosheet length. In another MD simulation performed by Jones *et al*.^50^, the Kapitza thermal resistance in aluminum/Gallium nitride bicrystal decreased by increasing the length. It can be explained that the predicted thermal conductivity by MD simulation depends on the system length along the heat flux direction, as the below equation predicts^42^:

3 $$\frac{1}{{K_{L} }} = \frac{1}{{K_{\infty } }}\left( {1 + \frac{\lambda }{L}} \right)$$

where $K_{\infty }$, *L*, and $\lambda$ refer to the thermal conductivity of the finite sample, the length of the nanosheet, and the phonon mean free path, respectively. As can be seen, the thermal conductivity increased against the length of the sample. The formula can be rewritten to. $K_{L} = K_{\infty } \left( {\frac{L}{L + \lambda }} \right)$ and subsequently placed in the equation of $J_{x} = K_{L} \frac{\Delta T}{L}$ so that the equation of $J_{x} = K_{\infty } \left( {\frac{\Delta T}{{L + \lambda }}} \right)$ would be obtained. The multiplication of $K_{\infty }$ and $\Delta T$ is a constant value. By substituting the $J_{x}$ in the $R_{k} = \frac{{\Delta T_{GB} }}{{J_{x} }}$, the relation between $R_{k}$ and the length of the sample is obtained as $R_{k} = \frac{{\Delta T_{GB} \left( {L + \lambda } \right)}}{{K_{\infty } { \times }\Delta T}}$. However, it is known that the $\Delta T_{GB}$ also changes with the changes of the length $\left( {\Delta T_{GB} \alpha L^{\beta } } \right)$. Therefore, according to the decreasing trend in the $R_{k}$ by an increase of the length, it is obvious that *β* has a value smaller than − 1.

## Conclusion

In the current work, the thermal properties of BC_3_NS-graphene hetero-nanosheets (BC_3_GrHs) having various types of grain boundaries [A_1_ (5–7–6–6–s), A_2_ (5–7–6–s), A_3_ (5–7–5–7–s), A_4_ (5–7–6–6–a), A_5_ (5–7–6–a), and A_6_ (5–7–5–7–a)] were studied through MD simulation. First, the temperature profile along BC_3_GrHs was plotted to verify the temperature drop in grain boundary (ΔT_GB_) at T = 300 K, ΔT = 40 K, and zero strain. It was observed that all temperature profiles showed discontinuity in the middle and ΔT_GB_ increased by increasing the defect density in grain boundaries so that the BC_3_GrHs having 5–7 and 5–7–5–7 defect pairs had the lower and higher ΔT_GB_, respectively. This happened due to the more phonon scattering in grain boundaries having higher defect concentration. Moreover, the temperature drop in asymmetric grain boundaries was higher than that of symmetric grain boundaries at the specified defect concentration. Next, the Kapitza resistance of mentioned grain boundaries and several parameters such as temperature and temperature gradient (ΔT) on its variation was investigated. It was revealed that Kapitza resistance increased by increasing the defect concentration in grain boundaries due to the higher phonon scattering rate in the grain boundary, which acted as a barrier in front of the heat flow and caused the increase in Kapitza thermal resistance. For example, A_1_ and A_3_ had the lowest (2 × 10^–10^ m^2^ K W^−1^) and highest (4.9 × 10^–10^ m^2^ K W^−1^) values of Kapitza resistance, respectively, among symmetric grain boundaries.

Moreover, the non-symmetric grain boundaries showed higher Kapitza resistance than the symmetric one at specified defect concentration. The temperature elevation caused the decrease of the Kapitza resistance due to increasing the energy of phonons which facilitated the heat flux along grain boundaries. The changes of the ΔT could not considerably affect the alteration of Kapitza resistance. The increase of strain caused the enhancement of Kapitza resistance due to a decrease in the phonon speed of atoms. The methodology implemented in the present work can be generalized to more complex nanostructures to predict and precisely adjust thermal properties of other heterostructures.
